# The Impact of Weaning Stress on Gut Health and the Mechanistic Aspects of Several Feed Additives Contributing to Improved Gut Health Function in Weanling Piglets—A Review

**DOI:** 10.3390/ani11082418

**Published:** 2021-08-17

**Authors:** Santi-Devi Upadhaya, In-Ho Kim

**Affiliations:** Department of Animal Resource and Science, Dankook University, No.29 Anseodong, Cheonan 31116, Choongnam, Korea; santi.upadhaya@gmail.com

**Keywords:** gut health, mechanistic aspect, nutritional intervention, piglet, weaning stress

## Abstract

**Simple Summary:**

The current review aimed to provide an overview on the problems associated with weaning with a special focus on gut health, and also highlighted the nutritional approach using different kinds of feed additives and their mechanistic aspects in mitigating production inefficiencies and gut health dysfunction in weanling pigs.

**Abstract:**

Newly weaned pig encounters psychosocial, physical, and nutritional stressors simultaneously when their immune system is not fully developed. These stressors have a cumulative effect on the immune response that contributes to the post-weaning growth lag which is characterized by depression in feed intake, reduced or negative growth rates, and increased susceptibility to pathogens in the first 24 to 48 h post-weaning. Consequently, the intestinal integrity, and digestive and absorptive capacity are impaired, and there is an increase in intestinal oxidative stress. It also causes the shifts in the taxonomic and functional properties of intestinal microbiome abruptly, thereby adversely affecting the health and performance of animals. It has been suggested that the effects of weaning stress on immune functions, intestinal barrier functions, and nervous system function in early weaned pigs extends into adulthood. The inclusion of different types of feed additives into the diet have been reported to alleviate the negative effects of weaning stress. The objective of this paper was to provide an overview on how the weaning stress affects gut health and the impact it has on production efficiencies, as well as the mechanistic aspects of several feed additives applied in reducing the weaning associated gut health problems and performance inefficiencies.

## 1. Introduction

In pigs’ lives, weaning is a rather challenging and stressful event that leads to enormous changes in the piglet’s gastrointestinal tract, resulting in the perturbations in gut microbiota, host physiology, and mucosal immune function [1] with subsequent reduction in feed intake, occurrence of post-weaning diarrhea, and growth reduction [2,3]. The mucosa epithelial cells are the largest immune organ in pigs and are the first responders to microorganisms in the gut. The health of the mucosa epithelium, the cells that line the gastrointestinal tract, plays a pivotal role on the growth and development of the pig through secretion and absorption. The intestinal mucosa is susceptible to inflammation since it is constantly exposed to the luminal environment including bacteria, toxins, and pathogens [4]. With the ban in the use of in-feed antibiotics due to rising public concerns of resultant antibiotic-resistant pathogens in both livestock and humans, the swine industry faces challenges of keeping pigs healthy, especially at post-weaning. Thus, the characteristic features of a healthy gut may include, but are not limited to, a healthy proliferation of epithelial cells lining the intestinal wall, proper gut barrier function, a beneficial and balanced gut microbiota, and a well-developed intestinal mucosa immunity [5]. Therefore, weaning pig requires adequate and high-quality nutrients, and proper husbandry and management practices, which are emphasized primarily towards rapid feed intake encouragement whilst reducing mortality and morbidity. Thus, to overcome the weaning transition problems, pre-weaning and post-weaning strategies are employed. Pre-weaning nutritional strategies aim to give piglets a stronger start before they encounter major stressors by supplementing creep feed [6]. However, post-weaning nutritional strategies aid piglet recovery post-weaning. This review is focused on describing post-weaning associated gut health issues and their impact on production efficiencies, as well as highlighting the effect of some selected feed additives in mitigating these problems.

## 2. Impact of Weaning Stress in Young Pigs

### 2.1. Gut Health and Intestinal Barrier

The gastrointestinal tract (GIT) is a very dynamic organ and is not only limited to digestion and absorption of nutrients but also maintains fluid balance, thereby achieving the required viscosity of luminal contents. It also secretes digestive enzymes, mucins, and immunoglobulins, and maintains barrier function against harmful pathogens and antigens [7,8]. The functioning of the GIT is highly influenced by a complex interaction between nutrition, the intestinal mucosal membrane, and the indigenous microflora which affect the entire physiology, health, and well-being of an animal [9]. Weaning transition leads to physiological change in structural and functional aspects of the intestine, leading to the atrophy of villous and increased crypt depth. These physiological changes can adversely affect digestion, absorption, secretion, and maintenance of barrier function, which may consequently lead to deprivation in feed intake, thereby reducing the growth performance of post weaning pigs [10,11,12]. For instance, Hampson [13] noted a reduction of villus height by 25 to 35% pre-weaning within 24 h in pigs that were weaned at 21 days of age, and the reduction in villous height continued for 5 days after weaning resulting in half of the initial height. Thus, for maximizing pig production, it is necessary to reduce physiological changes in the small intestine as much as possible during the weaning transition.

A single layer of columnar epithelial cells lining the intestinal tract functions as intestinal barrier. These cells are sealed by junctional complexes, including tight and adherens junctions, in close proximity to the apical and lateral sides of the paracellular space [7] and they provide the first line of defense against pathogenic microorganisms and antigens that are present within the intestinal lumen. The breakdown of the intestinal barrier results in increased intestinal permeability allowing the pathogenic agents present in luminal contents to leak across the epithelium, making it easy to access the sub-epithelial tissue [14,15]. This leakage consequently leads to inflammation, diarrhea, malabsorption, and systemic disease that can eventually influence animal health and growth status [8,16]. In several post-weaning swine enteric diseases, functional impairment of intestine has been shown to be the most important pathophysiological event. In a study, Moeser et al. [17] evaluated the intestinal dysfunction in pigs weaned at 19 days of age and found that weaned pigs had higher intestinal permeability and enhanced secretory activity in the jejunum and colon than in unweaned pigs which corroborated with the findings of Boudry et al. [10] who demonstrated a transient reduction in transepithelial resistance in jejunum. However, with the increase in the weaning age of the animal, there were improvements in intestinal barrier function as indicated by improved transient reduction in transepithelial resistance as well as reduction in mucosal-to-serosal flux of paracellular probes mannitol and inulin [18]. Earlier studies [19,20] suggest that disturbances in GI barrier functions and the immune and nervous system functions in early weaned pigs persist into adulthood. These findings suggest that weaning age can have an impact on the intestinal barrier function [17,18] and consideration should be taken to wean at an appropriate age for the healthy production of wean-finished pigs. Alleviating intestinal dysfunction during this process is important, given the direct relationship between animal health and economic productivity. Preserving intestinal health minimizes the adverse effects of weaning-induced stress.

### 2.2. Weaning Transition and Gut Microbiota

A dynamic composition of microbiota inhabits the GIT of pig which shifts over with time as well as along the different segments of GIT. Microbial colonization starts right from the time of birth and is shaped by the consumption of dam’s milk resulting in milk-oriented microbiome [21,22]. Weaning of pigs is done during weeks 3–4 in the modern swine industry, and pigs are fed solid diets [1]. This weaning transition is characterized by a shift in the microbial population where pathogenic bacteria increase in numbers [23]. The unhealthy alterations in gut microbiota composition triggered by weaning stress may be one of the major reasons for post-weaning diarrhea. It was revealed from a recent metagenomic analysis of the fecal microbiota that diarrhea was associated with increase in the relative abundance of *Prevotella*, *Sutterella*, *Campylobacter*, and *Fusobacteriaceae* [24].

Li et al. [1] showed remarkable differences in microbiome between nursing and weaning piglets. For instance, a reduction in *Alloprevotella* and *Oscillospira* whereas an increment in *Campylobacterales*, *Campylobacteraceae* and *Campylobacter* microbial population were observed in weaned piglets. It has been suggested that *Alloprevotella* mainly produce succinate and acetate, which plays a role in improving the gut barrier and exhibit anti-inflammatory function [25]. *Oscillospira* species are butyrate producers, and they can reduce the inflammatory disease condition [26,27]. The increase in *Campylobacteraceae* and *Campylobacter* after weaning indicates a life-threatening GIT disease [28]. In agreement with previous studies of Kim et al. [29] and Hu et al. [30], Li et al. [1] demonstrated that *Bacteroidetes* and *Firmicutes* were the two most dominant phyla in the intestine of piglets followed by *Proteobacteria* and *Fusobacteria,* regardless of weaning. However, in other studies, the relative abundances of microorganisms belonging to family *Bacteroidaceae* and *Enterobacteriaceae* declined over time, while there was a rise in the population of *Lactobacillaceae*, *Ruminococcaceae*, *Veillonellaceae*, and *Prevotellaceae* families in weaned piglets [31,32,33].

To deal with the stresses caused by dietary change during weaning transition, orientation of pig microbial community structure and functional capacities were notable. For example, *Prevotella* spp. has been reported to breakdown polysaccharides to short-chain fatty acids (SCFAs) through the production of enzymes, such as β-glucanase, mannase, and xylanase, which are capable of degrading plant cell wall [34,35]. In addition, the carbohydrates are metabolized by *Lactobacilli* and are fermented in the large intestine to SCFAs, which are finally utilized by the pigs as an energy source [36]. Thus, one of the major factors influencing abrupt shifts in the microbiota in piglets during weaning is the sudden alteration of diet from simple to more complex nutrient sources, which affects absorption capacity of the small intestine, and likely influences growth and feed efficiency.

### 2.3. Intestinal Mucosa Immunity and Oxidative Stress

The impacts of weaning stress are not limited to intestinal barrier function and gut microbiome but also observed in intestinal immunity and intestinal oxidative status of weaned pigs compared with pre-weaning pigs. The intestinal CD4+ and CD8+ T lymphocytes in pigs on day 2 post-weaning has been reported to increase sharply, thereby upregulating the mRNA expression of inflammatory cytokines such as tumor necrosis factor-alpha (TNF-a) and interleukins (IL-1b, IL-6, and IL-8) in the middle of jejunum [37,38], and a reduction in fecal immunoglobulin A (IgA) from day 5 after birth to 50 days of age was also reported [39]. In addition, the upregulation of matrix metalloproteinase through activation of immune cell and downregulation of major histocompatibility complex (MHC) class I expression in jejunal mucosa were observed in weaned pigs, resulting in atrophied villus and increased concentration of plasma cortisol [37,40]. These observations suggest that weaning induces a transient gut inflammation in pigs.

Increased oxidation processes due to weaning stress lead to the release of excessive reactive oxygen species which could eventually modify certain proteins in the cell and trigger the upregulation of pro-inflammatory cytokines, thereby negatively affecting the expression of tight junction proteins and causing increased gut permeability [41,42].

### 2.4. Feed Intake and Performance during Weaning Transition

As mentioned above, the functions of the GIT extend beyond digestion and the subsequent active or passive absorption of nutrients and electrolytes, barrier function, maintenance of bodily fluid balance, secretion of digestive enzymes, immunoglobulins, and multiple other components, as they also play an influential role in the regulation of epithelial and immune functions for normal biological functioning and homeostasis in both the GIT and the body [9,43]. After weaning at the age of 3–4 weeks, piglets have no choice other than adapting to a solid dry diet that is less digestible and palatable as compared with highly digestible and palatable dam’s milk in the liquid form pre-weaning. Apart from this nutritional challenge, weaning is simultaneously involved in social and environmental stressors, consequently leading to a low feed intake [44], reduced growth, and behavioral disturbances [45] along with gastrointestinal problems [23,46]. In a review by Dividich and Seve [44], it has been indicated that the intake of metabolizable energy (ME) is reduced by 30–40% of pre-weaning milk intake, and to achieve full recovery to the pre-weaning ME intake level, it takes approximately 2 weeks post-weaning. This low feed intake during the post-weaning period may contribute to intestinal inflammation adversely affecting intestinal integrity resulting in the reduction of villus height and an increase in crypt depth [37]. It is obvious that growth performance is reduced with low feed intake. For instance, a loss of 100–250 g body weight (BW) is reported at the first day of weaning regardless of weaning age, which is recovered by 4 days post-weaning [44]. The BW gain above 227 g/day during the first week after weaning led to the reduction in days to market by 6–10 days compared to BW gain by less than 150 g/d during the first week, indicating the days of weight gain in the first week after weaning impacts the total days to market [47]. Thus, it is of utmost importance to enhance the feed consumption and growth in weaned pigs as soon as possible. Although it is difficult to prevent some of the reduction in BW during weaning transition, it is necessary to understand the impact of reduced feed intake due to weaning stress and its concomitant impact on performance and take appropriate measures to reduce the negative effects.

For the improvement of nutrient digestion and absorption, regulation of gut microbiota, as well as modulation of immune system so as to enhance disease resistance and nutritional intervention along with management techniques have been considered as a good strategy [5,6,48,49,50].

## 3. Nutritional Intervention and Mechanistic Aspects

Nutritional strategies, such as optimizing dietary proteins or energy content and the use of feed additives at post-weaning, have been implemented to minimize the weaning-induced stress. In an era of reduced/banned antibiotic growth promoters, the supplementation of different feed additives such as probiotics, prebiotics, organic acids, plant extracts, short-chain fatty acids, polyunsaturated fatty acids, etc. have been shown from various studies to beneficially influence growth performance and the compromised state of gut health of the young pig after weaning (Table 1). For conducting this review, a literature search was performed using PubMed, Science Direct, Google scholar, and Web of Science databases. Data collection was performed based on 148 articles published during the years 1986–2021. Ziegler et al. [51] pointed out that the fundamental physiologic, anatomic, and nutritional similarities exist between pigs and humans, and both share similar gut microbial profiles [52].

Therefore, certain aspects of work can be applied to pigs, particularly the mechanistic studies focused on the interaction between certain additives and the host mucosal surface or pathogenic bacteria.

### 3.1. Nucleotides

Nucleotides are a group of bioactive agents that take part in building DNA or RNA, in various biochemical processes, biosynthetic pathways, and coenzyme components and are absorbed as nucleosides by the intestinal epithelium [80,81,82]. Research has shown that exogenous supplementation of nucleotides induces positive effects on intestinal hyperemia, immune response, small intestinal growth, intestinal microbiome, and hepatic composition in pigs [83,84,85,86], as well as increased BW and average daily gain (ADG) in weaning pigs [57]. Sauer et al. [81] noted that dietary nucleotides supplementation to single stomached animals positively affected nutrient metabolism, intestinal morphology, and function, immune function, intestinal microbiota, as well as growth performance. A recent study by Jang and Kim [55] demonstrated that nucleotide supplementation reduces intestinal inflammation and oxidative stress and improves intestinal villi structure and energy digestibility. Moreover, there is a high need of nucleotide especially during periods of growth, stress, and immunodeficiency in newly weaned pigs [87] since piglet starter diets are nucleotide deficient [88]. The in vivo feeding trial performed by Lee and Kim [89] in weaned pigs showed that nucleotide supplementation resulted in enhanced growth performance and intestinal morphology as well as reduction in serum stress levels which corroborated with findings of previous studies [81,90] which demonstrated that dietary nucleotide, when supplemented at greater than physiological quantities, improved growth performance and plasma cortisol levels as well as enhanced the adaptive capabilities of piglets to weaning stress during the first 2 weeks after weaning.

Feeding a diet supplemented with nucleotide to low-birth-weight pigs has been reported to markedly increase gene expressions of Toll-like receptors (TLR-9, TLR-4) and Toll-interacting protein (TOLLIP), indicating the effect of nucleotides supplementation on eliciting intestinal innate immunity and mounting acquired immune response [82]. In addition, supplementing nucleotides led to the upregulation of tight junction proteins such as Claudin-1 and ZO-1 in ileum of piglets [82] as well as Claudin-3 and E-cadherin expression, and the pyrimidine nucleotide metabolic enzymes in the duodenal mucosa [56] which may eventually aid in alleviating gut health problem by improving intestinal barrier functions in weanling pigs.

The effects of exogenous nucleotide during the weaning transition were evaluated using gene expression profiling of the small intestine of pigs after dietary treatment with nucleotides by Lee and Kim [89]. Genes that were significantly regulated by nucleotide were identified and further study was conducted to assess the regulatory functions for small intestinal development in pigs. Among the top 10 upregulated genes, Trefoil factor 3 (TFF3) and SAM-pointed domain-containing ETS transcription factor (SPDEF) were found to have a significant role in wound healing and intestinal barrier function. In addition, when lipopolysaccharide-challenged IPEC-J2 intestinal porcine enterocyte cells were treated with nucleosides and TFF3, it resulted in increased intestinal trans-epithelial electrical resistance and decreased intestinal permeability. Finally, Lee and Kim [89] showed that nucleotide treatment induced the expression of SPDEF in a dose-dependent manner, resulting in modulation of TFF3-mediated wound healing and intestinal barrier function via the phosphatidylinositol 3-kinase/Akt, extracellular signal-regulated kinase 1/2, p38, and Janus kinase/signal transducer and activator of transcription signaling pathways.

### 3.2. Phytogenic Compound

**Figure 1:**
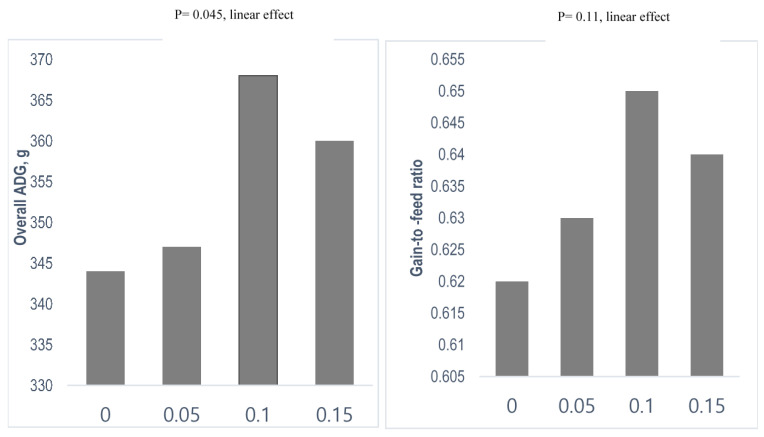
Effect of dietary supplementation of increasing doses of *E. cava* (%) on overall average daily gain (ADG) and gain-to-feed ratio (G:F) in weaning pigs.

The effects of phytogenic feed additive on monogastric animals’ performance and health have been thoroughly reviewed by Upadhaya and Kim [91] and Lillehoj et al. [92]. Different plant resources such as garlic, pepper, cinnamon, clove, fennel, oregano thyme, ginger, turmeric, rosemary, caraway, etc. enriched in bioactive phytochemicals have been reported to possess antimicrobial, anti-inflammatory, as well as antioxidant properties. These bioactive phytochemical compounds including allicin, capsaicin, eugenol, anethol, carvacrol, thymol, cinnamaldehyde, curcumin, etc. play an influential role in enhanced disease resistance and growth performance. The observed beneficial effects can be associated with improved gut health, such as improved intestinal barrier integrity [93,94], due to the upregulation of genes such as MUC2 and genes encoding claudins and occludins related to tight junctions and cell to cell junction in ileum. Yuan et al. [95] reported that by the dietary inclusion of flavones extracted from the leaves of *Eucommia ulmoides* enhanced intestinal morphology and integrity of diquat challenged pigs via improved intestinal barrier function. In addition, several other studies reported that supplementation of plant extract alone or in combination improved performance, digestibility, and intestinal barrier function in weaning pigs [77,78,79]. *Ecklonia species* (commonly known as brown algae) possessing a wide range of therapeutic properties, and enriched with vitamins, minerals, dietary fiber, proteins, and polysaccharide have received considerable attention in recent decades. The beneficial effects of increase in the dietary supplemental level of *Ecklonia cava* (0%, 0.05%, 0.1%, 0.15%), consisting of phloroglucinol, eckol, phlorofucofuroeckol, and dieckol, were observed in cecal microflora and intestinal morphology. In addition to this, a linear increase in overall average daily gain and feed efficiency (Figure 1) in weaning pigs has been reported by Choi et al. [96]. To evaluate the molecular mechanism of how eckol mitigate intestinal dysfunction, the changes in gene expression and intestinal function after Eckol treatment during the suckling-to-weaning transition were evaluated by Lee and Kim [79]. These authors investigated the biological roles of differentially expressed genes (DEGs) in intestinal development by assessing intestinal wound healing and barrier functions, as well as the associated signaling pathways and oxidative stress levels. An in vivo trial with eckol in weaning pigs altered the gene expression in intestinal samples and the expression patterns were confirmed in the small intestine. Furthermore, according to Lee and Kim [79], treating the IPEC-J2 intestinal porcine enterocyte cell line with different concentrations ranging from 10 to 200 μM of eckol and incubating for 24 h resulted in a concentration-dependent increase in pancreatic and duodenal homeobox (PDX)1, and heparin-binding EGF-like growth factor (HBEGF) mRNA and protein levels. Additionally, eckol alleviated H_2_O_2_-induced oxidative stress through PI3K/AKT, P38, and 5′-AMP-activated protein kinase (AMPK) signaling pathways. Thus, eckol was found to be a potential candidate in modulating intestinal barrier functions, wound healing, and oxidative stress through PDX/HBEGF, thereby improving growth during the suckling-to-weaning transition.

### 3.3. Probiotics

The non-pathogenic, viable microorganisms possessing the ability to reach the intestines in sufficient numbers and conferring beneficial impact on host are termed as probiotics [97,98]. *Lactobacillus* species, *Bifidobacterium* species, *Escherichia coli* (*E. coli*), *Bacillus* species, and *Saccharomyces* species are the commonly used probiotics either alone or in combination. Several studies have shown that different probiotics strains’ application beneficially impacted intestinal integrity, fecal microbial counts, enhanced gut health, and improved performance in weaning pigs [69,70,71,72,73,74,75,76]. In addition, our previous study showed that Salmonella-challenged weaning pigs, when fed diet supplemented with 0.1% *B. subtilis* RX7 containing 1 × 10^9^ cfu/g or *B. methylotrophicus* C14 containing 1 × 10^9^ cfu/g, exerted positive immunomodulatory effects [99]. The major functions of probiotics include competitive adherence to the mucus and epithelium, enhanced intestinal barrier function, as well as immunomodulatory effects [100,101]. The abatement of barrier disruption by certain *Lactobacillus species* is through upregulation of tight junction proteins. For instance, an increase in occludin protein expression has been reported by the use of *L. acidophilus* and *L. plantarum* [102,103]. In addition to this, apical relocalization of ZO-1 and occludin through stimulation of Toll like receptor 2 has been reported to be induced by *L. plantarum* [104,105]. The preservation of barrier function by *Bifidobacterium* has been suggested to be due to the maintenance of tight junction confirmation [106]. Another reported mechanism of probiotics to improve barrier function and exclusion of pathogens is due to enhancement in mucin expression. In human cell lines Caco-2 (MUC2) and HT29 (MUC2 and 3), several *Lactobacillus* species have been reported to increase mucin expression, thereby blocking pathogenic *E. coli* invasion and adherence [107,108]. Despite the promising effects of probiotics on intestinal barrier homeostasis and repair, the potential drawbacks and limitations of probiotic therapy cannot be ignored. For instance, Shanahan [109] highlighted the potential risks of probiotic since no probiotic can be regarded with zero risk and noted that the adverse effect of a probiotic product depends on the safety of the product, physiological state, and susceptibility of host.

### 3.4. Prebiotics

The indigestible food ingredients’ ability to selectively stimulate the proliferation and activity of beneficial microorganisms, thereby benefiting the host, is known as prebiotics [110]. The beneficial effects of several prebiotics such as fructo-oligosaccharide, levan-type fructan, inulin, lactulose, galacto-oligosaccharide, and resistant starch on weanling pigs have been reported by several studies [63,64,65,66,67,68]. The proposed mechanisms of action of many prebiotics are due to the enhancement of the intestinal barrier function via the modulation of intestinal tight junction [111]. In in vivo studies by Wang et al. [112,113] on suckling piglets and LPS-challenged mice, it was demonstrated that galacto-oligosaccharide pretreatment led to the upregulation of ZO-1, occludin, and claudin-1 gene expression. In an in vitro study, a significant upregulation of tight junction genes including occludin, claudin-3, and ZO-1 was observed with the supplementation of inulin fermentation products to porcine intestinal epithelial cells [114]. The supplementation with fructo-oligosaccharides and butyrate to T84 human colonic epithelial cells and Caco-2 cells, respectively, resulted in the redistribution of proteins, including ZO-1 and occludin, to the vicinity of the tight junctions [115,116]. A reasonable explanation for these observed changes in tight junction protein expression and distribution is due to the direct effects of prebiotic on gut microbiota. As revealed from different studies in conjunction with the changes mentioned above in the epithelial barrier, prebiotic supplementation also results in robust activation of AMP-activated protein kinase (AMPK) [115,116,117] which may eventually have a significant modulatory effect on intestinal tight junction proteins.

### 3.5. Fatty Acids

Based on their carbon chain length, fatty acids are classified into short-chain fatty acids (SCFAs; 1–5 carbon atoms), medium-chain fatty acids (MCFAs; 6–12 carbon atoms), or long-chain fatty acids (LCFAs; 13–21 carbon atoms). In addition to this, fatty acids are classified into saturated or unsaturated fatty acids according to saturation level. These fatty acids possess bacteriostatic or bactericidal properties and are used as a pig feeding strategy.

**Figure 2:**
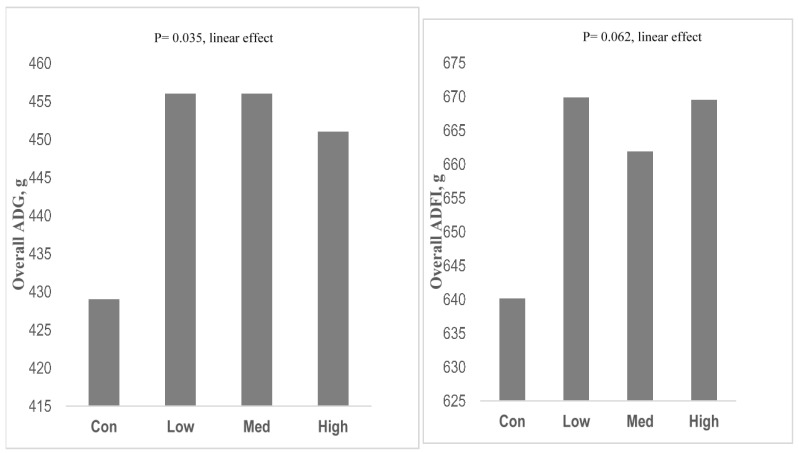
Effect of dietary supplementation of increasing dose of coated sodium butyrate acid on the overall average daily gain (ADG) and average daily feed intake (ADFI) of weaning pigs. Con, basal diet (lactose and sodium reduced); Low, Con + 0.5 kg/ton coated sodium butyrate; Med, Con + 1.5 kg/ton coated sodium butyrate and after 3 weeks Con + 0.75 kg/ton; High, Con + 3.0 kg/ton coated sodium butyrate and after 3 weeks Con + 1.50 kg/ton.

The SCFA such as butyrate supplementation in salt form (sodium n-butyrate) in weaning pigs has been reported to promote the performance traits in weaning pigs; inhibit the proliferation of pathogenic bacteria; and enhance nutrient digestion, absorption, and gut barrier function of piglets [118,119,120]. A recent study by Upadhaya et al. [121] also showed that the dietary supplementation of coated sodium butyrate at low and medium doses to lactose- and sodium-reduced diets improved overall ADG, and a low dose tended to improve ADFI in weaning pigs (Figure 2). In addition, this coated sodium butyrate improved villus height and the small intestinal microflora, suggesting its role in enhancing gut health.

Fatty acids exert inhibitory effects against microorganisms, but the minimum inhibitory concentrations differ depending on fatty acid types as well as the types of microorganisms and environmental pH. The concentration of SCFA increases under low pH, and can easily pass into the bacterial cells because of their higher intercellular pH, consequently causing the dissociation of SCFA and thereby reducing the intracellular pH of microbial cells and subsequently changing the metabolism of bacterial cells [122].

The MCFAs and monoglycerides are antimicrobial agents with the ability to disrupt the phospholipid membrane surrounding the pathogens [123]. The anionic part of MCFAs has been suggested to produce a strong antibacterial effect resulting in the alteration of the physico-chemical characteristics of the GIT environment in which the microorganisms exist, thereby influencing the gene expression of microorganism and host. The inhibitory activity against Gram-positive bacteria is stronger than Gram-negative bacteria because of the variation in the structural make-up of the cell wall of Gram-positive and Gram-negative bacteria. A simpler, single lipid bilayer cell membrane structures is found in Gram-positive bacteria, whereas Gram-negative bacterial have more complex inner and outer membrane structures [124]. Previous studies reported that monoglyceride, lauric acid, and blends of caprylic and capric acids exhibited antibacterial activity against several bacterial pathogens in swine [125]. Earlier studies have also shown that MCFA supplementation resulted in improved performance, enhanced nutrient digestibility, and improved antioxidant capacity of weaned piglets [126,127]. In an in-vivo study by Lee and Kang, [128], it was demonstrated that the supplementation of 0.5% capric acid resulted in reduced oxidative stress and improved intestinal barrier function in miniature pigs with cyclophosphamide-induced intestinal inflammation, oxidative stress, and gut barrier function. In addition, in an in vitro cellular model, Wang et al. [129] reported that caprylic acid supplementation resulted in enhanced intestinal epithelial barrier function due to the increased expression of endogenous host defense peptides, such as β defensin [129,130].

Several studies in human have reported the broad spectrum of antibacterial activity of polyunsaturated fatty acids [131,132,133]. Their mechanism of action has been suggested to be due to the obstruction in the essential bacterial processes at the pathogen membrane level through the disruption of electron transport chain, uncoupling of oxidative phosphorylation, and cell lysis [134,135]. A study by Zhang et al. [54] reported that supplementation of coated omega-3 fatty acid at the doses of 5, 10, and 15 g/kg showed trends in linear reduction in fecal *E. coli* counts and increments in *Lactobacillus* counts, suggesting that omega-3 fatty acid can also modulate gut microbials and enhance gut functions. In another study, Hanczakowska et al. [53] reported that supplementation of 2 g/kg capric or caprylic acid improved performance and mucosal epithelium structure of ileum in weaning pigs. However, there are also reports in which the supplementation of omega-3 fatty acid derived from linseed and coated docosahexaenoic acid supplementation derived from fish oil did not have a detectable impact on fecal microbiota in growing and weaning pigs, respectively [136,137]. More studies are needed to confirm the antimicrobial activity of PUFA in weaning pigs.

### 3.6. Organic Acids

With the existing evidence on the antimicrobial properties of some organic acids through the modulation of microbiota populations and reduction of pathogenic bacteria, organic acids are no longer regarded as simple acidifiers of animal feed, but rather as growth promoters and potential antibiotic substitutes [58,59,60,61,62,138,139,140,141,142,143]. For instance, lactic acid supplementation stimulated pancreatic secretion of piglets and reduced the incidence of post-weaning diarrhea [58,138]. In contrast, the microbiota composition in the gastrointestinal tract of weanling pigs, or the *E. coli* count in post-weaning pigs were not affected by citric acid supplementation [139,140]. However, the combination of organic acids (0.416% fumaric and 0.328% lactic acid) and medium-chain fatty acids (0.15% capric and caprylic acid) has been demonstrated to modulate gut microbiota and prevent post-weaning diarrhea [141]. In another study, Ferrara et al. [142] reported that the organic acid blends (0.15% caprylic and capric acids + 0.41% fumaric acid and 0.32% lactic acid) with and without MCFA increased the number of intra-epithelial lymphocytes in the jejunum. Ahmed et al. [143] also indicated that supplementation of 0.4% acidifier blend consisting of 17.2% formic acid, 4.1% propionic acid, 10.2% lactic acid, 9.5% phosphoric acid, and SiO_2_ 34.0% in the basal diet of weaned piglets led to the reduction in fecal counts of pathogenic gram-negative *Salmonella* and *E. coli* and increased beneficial *Lactobacilli* and *Bacilli* concentrations compared to the control. Our recently published study investigated the effects of blends of 40% coated organic acid blends (17% fumaric acid, 13% citric acid, 10% malic acid), 1.2% MCFAs (capric and caprylic acids), and 58.8% vegetable oil carriers on the growth performance of weaning pigs. When this feed supplement was incorporated into the diet at a 0.2% dose, it led to improvements in growth performance and nutrient digestibility [60].

The antimicrobial properties of organic acids are suggested to be due to their ability to cross the bacterial cell membrane. Organic acids are considered as bioactive compounds when their minimum inhibitory concentration is equal to or lower than 1000 ppm [144]. Organic acids in their undissociated form can modify the proton and associated anion concentrations in the cytoplasm, thereby negatively affecting essential enzymes and purine bases, resulting in the reduction of bacterial viability [145].

Thus, it can be seen that using blends rather than single organic acids, and coated versus non-coated organic acids tended to have wider-ranging action against pathogens in improving gut health and performance. However, some studies did not observe antimicrobial activities of certain organic acid feed additives either in coated or non-coated forms [146,147,148], suggesting that further studies are needed in order to optimize concentrations, combinations, and interactions of these compounds against target pathogens.

## 4. Conclusions

Weaning is one of the most stressful and complex events in pigs’ lives. Among several stressors, the stress of separation of piglets from their dam and abrupt change in the diet of piglets from the liquid to solid diet is quite critical, leading to gut-associated problems and immune functions which may persist until adulthood. The intention of this paper was to review the current knowledge in the literature on the nutritional intervention and the mechanistic aspects of several feed additives such as probiotics, prebiotics, organic acids, plant extracts, short-/medium-chain fatty acid, and polyunsaturated fatty acid in mitigating the impact of weaning stress on the gut health of weaning pigs. Moreover, there are many more candidates of feed additives than the ones listed in this review that are influential in regulating intestinal environments and enhancing weaned pig performance. The mechanistic aspect of different feed additives mentioned in this review in alleviating intestinal dysfunction is basically due to the bacteriostatic or bactericidal properties of these feed additives against the pathogens or due to the change in the expression of certain genes related with tight junctions influencing certain signaling pathways or stimulation of certain gastric cells and enzymes that eventually promote gut health. Based on the published literatures, it can be seen that these feed additives exert positive effects as well as non-significant effects in growth performance and gut health. The reported positive impact of these feed additives indicate that these feed additives can be effectively used to support a profitable and sustainable swine production. However, due consideration must be given to the dose, efficacy, and safety on the usage of these feed additives. The non-detectable positive impact of some of these feed additives could have been influenced by the energy content and protein level in the diets of young pigs and thus consideration should be taken on the diet composition. More research into optimizing these feed additives and the corresponding feeding regimen is suggested.

## Figures and Tables

**Table 1:** Effects of different types of feed additives on gut health and performance of young pigs.

Additive Type	Composition	Age of Piglets/Experiment Duration	Dose	Intestinal Structure, Gut/Health Microbiota	Performance/Other Observations	References
Fatty acid (FA)						
Medium-chain fatty acid	Caprylic or capric acid	32 days old/49-d trial	2 g/kg	Reduced *Clostridium perfringens* levels;improved mucosal epithelium structure of ileum	Improved overall ADG. FCR in pigs receiving diet supplemented with caprylic acid was better; increased digestibility and reduced mortality	[53]
Poly unsaturated fatty acids	Coated n-3 FA	28 days old/42-d trial	5, 10 and 15 g/kg.	Trends in linear increment in fecal Lactobacillus counts at weeks 3 and 6	Linear increase in ADG during week 1, 3 and overall, G:F linearly increased during overall; linear increase in DM and N digestibility at the end of experiment	[54]
Nucleotides						
	UMP, GMP, AMP, CMP, and IMP	19 days old/21-d trial	0, 50, 150, 250, and 500 mg/kg	Quadratic response on villus height–crypt ratio, linear reduction in crypt cell proliferation in jejunum, reduced jejunal IL-6 when nucleotide increased from 50 to 150 mg/kg	Increased ADG during the first 11 days when pigs received diet supplemented with 50–150 mg/kg nucleotide	[55]
	UMP/UR	12 days old/10-d trail	476 mg UP, 348 mg UR(orally)	Decreased the diarrhea rate, UR increased the jejunum villus length/crypt depth ratio, Claudin-3, and E-cadherin expression, and the pyrimidine nucleotide metabolic enzymes in the duodenal mucosa, UMP and UR decreased the expression of CAD and RRM2 at the jejunal mucosa	UMP and UR supplements improved the ADG of piglets	[56]
	UMP, GMP, AMP, CMP, and IMP	28 days old/28-d trial	0.8 g/head/day	No differences in gene expression levels of inflammatory cytokines (IL1α, IL1β, IL6, IL10, TNFα, TLR2, TLR4, and PPAR) at ileal Peyer’s patches level; no effect on IgA and IgG content in blood	Increased BW and ADG but not G:F	[57]
Organic acid (OA)						
	Pure OA (propionic acid, lactic acid, formic acid, malic acid, citric acid, or fumaric acid)	25 days old/28-d trial	10, 16, 12, 12, 15, and 15 g/kg, respectively	Reduced incidence and severity of diarrhea	Heavier BW, and increased ADG and FI especially with lactic acid supplementation	[58]
	Formic acid	35 days old/42-d trial	0, 1.4 g/kg (low formic acid; LFA), or 6.4 g/kg (high formic acid; HFA)	Increased microbiota diversity in high FA group	Increased ADG, ADFI and improved feed efficiency during the first three weeks in both high and low OA group	[59]
	17% fumaric acid, 13% citric acid, 10% malic acid, and 1.2% medium-chain fatty acid (protected OA)	28 days old/42-d trial	1 and 2 g/kg	Reduction in *E. coli* counts at week 3 and increase in *Lactobacillus* counts in week 6 with 2 g/kg organic acid	Increased overall ADG and ADFI	[60]
	Formic acid, acetic acid, and propionic acid combined with medium chain fatty acids	28-d trial	3 g/kg	Reduced the incidence of diarrhea and fecal *E. coli* counts, the ratio of villus height-to-crypt depth in the jejunum and ileum was higher	Digestibility of dry matter, total carbohydrates, NDF, and ADF was increased during days 14–28	[61]
	17% fumaric acid, 13% citric acid, 10% malic acid, and 1.2% medium-chain fatty acid	28 days old/42-d trial	0, 1, and 2 g/kg	Linear increase in fecal *Lactobacillus* counts and linear reduction in *E. coli* and Salmonella counts	Increase in overall ADG and DM digestibility	[62]
Prebiotics						
	Fructo-oligosaccharide	33 days old/21-d trial	4 g/kg	Increased villus height, reduced diarrhea	Improved ADG, increased the concentrations of isobutyric and butyric acid and total VFAs in the caecum, and acetic acid, isovaleric acid, and total VFAs in feces	[63]
	Resistant starch	17 days old/21-d trial	70 and 140 g/kg	Enhanced microbial diversity in colon and reduced diarrhea with 7% resistant starch inclusion in the diet	No effect on growth performance	[64]
	Inulin	42 day old/35-d trial	40 mg/kg	Increased *Lactobacilli* and *Bifidobacteria* and reduced *Enterobacteriaceae* and *Clostridium* spp. in the lumen and mucosa of gut	Higher blood hemoglobin	[65]
	Lactulose	25 days old/18-d trail	10 g/kg	Increased *Lactobacilli* and the percentage of butyric acid in the colon; an increase in the ileum villous height	Improved the ADG; reduction of the pig major acute-phase protein in serum	[66]
	Levan-type fructan	28 days old/42-d trial	0, 0.1, 0.5, and 1.0 g/kg	Linear increase in fecal lactic acid bacteria counts with the increase in the dose of levan	Increased ADG and ADFI linearly during days 0–21 and overall; linear increase in the digestibility of DM, CP, and GE	[67]
	Galacto oligosaccharide (GOS)	28 days old/28-d trial	0, 500, 1000, 1500, and 2000 mg/kg	Increased the number of *Lactobacillus* and *Bifidobacterium*, and decreased the number of *E. coli* in a linear or quadratic dose-dependent manner; decreased serum concentration of pro-inflammatory cytokines but increased anti-inflammatory cytokines in a linear or quadratic dose-dependent manner	Promoted the growth and activities of antioxidant enzyme in a linear or quadratic dose-dependent manner	[68]
Probiotics						
	*Saccharomyces cerevisiae*	27 days old/35-d trial	1.25 g/kg	Villus length and crypt depth not affected by probiotic but were greater at 5 weeks vs. 2 weeks after weaning; CD4 and CD8 cells were lower at 5 week after weaning	Improved ADG and G:F	[69]
	*Lactobacillus sobrius*	21 days old (challenged with 1.5 mL suspension of 10^10^ CFU ETEC F4)	10^10^ CFU probiotic in 1 mL skimmed milk/day	Reduced ETEC levels in the ileum	Improved daily weight gain	[70]
	*Saccharomyces cerevisiae*	21 days old/21-d trial	5, 10, 20 g/kg probiotic	Increase in jejunal villus height and villus height: crypt depth ratio was also increased; gut IFN-gamma concentration increased on day 21 but plasma IFN-gamma reduced on day 7 and CD4 reduced on day 14	Feed intake was enhanced with the inclusion of 5 or 10 g/kg; enhanced digestibility of DM, CP, GE with 5 g/kg yeast supplement	[71]
	*Lactobacillus johnsonii* and *Lactobacillus mucosae* single or combined	21 days old/21-d trial	5 × 10^7^ or 10^8^ CFU/g/piglet/day of each strain	Increase in fecal *Lactobacillus* and reduction in *E. coli* counts	Both single or combined supplementation increased overall FI and BWG	[72]
	*Enterococcus faecalis*	24–26 days old/28-d trial	0.5 × 10^9^, 1 × 10^9^, or 2.5 × 10^9^ CFU/kg of feed	Lower incidence of diarrhea and increase in fecal *Lactobacillus* counts	Higher ADG and feed efficiency in pigs receiving the highest probiotic dose.	[73]
	*Bacillus subtilis* GCB-13-001 (1 × 10^9^ CFU/kg)	28 days old/42-d trial	1 g/kg	Fecal *Lactobacillus* counts were improved, and *E. coli* counts were reduced	The BW and ADG improved during all phases, F:G improved during the overall experiment period	[74]
	*B. coagulans, B. licheniformis, B. subtilis,* and *C. butyricum* mixed dried spores(1 × 10^12^, 5 × 10^11^, 1 × 10^12^ and 1 × 10^11^ CFU/kg respectively).	28 days old/42-d trial	0, 1, 2, and 3 g/kg	Linear increase in fecal *Lactobacillus* counts and decreased *Escherichia coli* counts and ammonia (NH3) emission	Linearly increased ADG and ADFI during d 0–7, increased ADG and G:F during d 8–21	[75]
	*Bacillus subtilis* or *Bacillus pumilus* 1 × 10^9^ CFU/kg	21 days old (challenged with ETEC/28-d trail)	500 mg/kg	*Bacillus subtilis* supplement alleviated diarrhea severity, enhanced gut health, and reduced systemic inflammation of weaned pigs infected with ETEC F18	*Bacillus subtilis* supplementation improved growth performance	[76]
Phytogenics						
Mixture of herb extract	Buckwheat, thyme, curcuma, black pepper, and ginger	21 days old/42-d trial	250 mg/kg	Reduced fecal *E. coli* counts	Improved energy digestibility but no effect on growth performance	[77]
Plant extract(PE)	Green tea leaves (*Camellia sinensis*) and pomegranate fruit (*Punica granatum*)	24 days old/35-d trial	8 μL/kg per day PE in drinking water	Reduced fecal *E. coli* counts in challenged pigs on days 14 and 35 and reduced *Enterobacteriaceae* on day 35	Increased ADG from days 28–35 and increase G:F ratio from days 7 to 14	[78]
Brown algae (ethanol extract from Ecklonia sp.)	Eckol	28 days old/42-d trial	0.5 and 1 g/kg	Improved intestinal barrier function	Improved growth performance, and reduced the levels of stress hormones (cortisol, epinephrine, and norepinephrine) and antioxidants (superoxide dismutase and glutathione peroxide)	[79]

Abbreviations: ADG, average daily gain; ADFI, average daily feed intake; ADF, acid detergent fiber; CP, crude protein; DM, dry matter; ETEC, enterotoxigenic *Escherichia coli*; FCR, feed conversion rate; G:F, gain-to-feed ratio; GE, gross energy; N, nitrogen; NDF, nitrogen detergent fiber; VFA, volatile fatty acid; UMP, uridine 5′monophosphate, GMP, guanosine 5′monophosphate; AMP, adenine 5′monophosphate; CMP, cytidine 5′monophosphate; IMP, inosine 5′monophosphate; UR, Uridine.

## Data Availability

Not applicable.
